# Peanut and cotton intercropping increases productivity and economic returns through regulating plant nutrient accumulation and soil microbial communities

**DOI:** 10.1186/s12870-022-03506-y

**Published:** 2022-03-16

**Authors:** Wei Xie, Kai Zhang, Xiaoying Wang, Xiaoxia Zou, Xiaojun Zhang, Xiaona Yu, Yuefu Wang, Tong Si

**Affiliations:** Shandong Provincial Key Laboratory of Dryland Farming Technology, College of Agronomy, Qingdao Agricultural University, Qingdao, 266109 P.R. China

**Keywords:** Below-ground interactions, Economic returns, Intercropping, Saline soil

## Abstract

**Background:**

Intercropping (IC) has been widely adopted by farmers for enhancing crop productivity and economic returns; however, the underpinning mechanisms from the perspective of below-ground interspecific interactions are only partly understood especially when intercropping practices under saline soil conditions. By using permeable (100 μm) and impermeable (solid) root barriers in a multi-site field experiment, we aimed to study the impact of root-root interactions on nutrient accumulation, soil microbial communities, crop yield, and economic returns in a peanut/cotton IC system under non-saline, secondary-saline, and coastal saline soil conditions of China.

**Results:**

The results indicate that IC decreased the peanut pods yield by 14.00, 10.01, and 16.52% while increased the seed cotton yield by 61.99, 66.00, and 58.51%, respectively in three experimental positions, and consequently enhanced the economic returns by compared with monoculture of peanut (MP) and cotton (MC). The higher accumulations of nutrients such as nitrogen (N), phosphorus (P), and potassium (K) were also observed in IC not only in the soil but also in vegetative tissues and reproductive organs of peanut. Bacterial community structure analysis under normal growth conditions reveals that IC dramatically altered the soil bacterial abundance composition in both peanut and cotton strips of the top soil whereas the bacterial diversity was barely affected compared with MP and MC. At blossom-needling stage, the metabolic functional features of the bacterial communities such as fatty acid biosynthesis, lipoic acid metabolism, peptidoglycan biosynthesis, and biosynthesis of ansamycins were significantly enriched in MP compared with other treatments. Conversely, these metabolic functional features were dramatically depleted in MP while significantly enriched in IC at podding stage. Permeable root barrier treatments (NC-P and NC-C) counteracted the benefits of IC and the side effects were more pronounced in impermeable treatments (SC-P and SC-C).

**Conclusion:**

Peanut/cotton intercropping increases crop yield as well as economic returns under non-saline, secondary-saline, and coastal saline soil conditions probably by modulating the soil bacterial abundance composition and accelerating plant nutrients accumulation.

**Supplementary Information:**

The online version contains supplementary material available at 10.1186/s12870-022-03506-y.

## Background

Intercropping has been defined as simultaneously cultivation of two or more crop species in close proximity [1–3]. Generally, intercropping system has been widely adopted by farmers for it shows a positive relationship between plant diversity and agricultural productivity worldwide [4–6]. Compared with monocropping systems, the advantages of intercropping in over-yielding have been explained by niche complementarity and interspecific facilitation [7–9]. In a cropping system, the interspecific facilitation might come from the above-ground and below-ground parts of the crops. Literatures indicated that planting arrangements e.g. plant density and strip width achieve a dominant position in controlling the strength of interspecific crop interactions and yields [10–12]. An earlier report indicated that cotton/peanut intercropping system could increase the crop productivity through regulating the photosystem and the maximum leaf area index of peanut [13, 14]. These studies documented the profound role of the above-ground organs in the interspecific facilitation. Alternatively, from the perspective of the below-ground part of the crops, the interspecific facilitation may occur via transfer of nutrients through co-cultivated crop species or stimulation of beneficial soil microbes as affected by root exudates [15–18]. Although intercropping has been proved to be an efficient land use and sustainable agricultural practice that is widely practiced worldwide, knowledge on this intercropping system is still lacking from the perspective of saline growth conditions [19, 20].

Salinity is one of the major abiotic stresses and has become an ever-increasing threat to agricultural production around the globe [21–24]. It is estimated that over 50% of the arable land on our planet will be salinized by the middle of this century [25, 26]. Crop species show great variability in their inherent saline tolerance. Plants have evolved sophisticated physiological mechanisms to cope with salinity [27–29]. Upon a salinity stress, the first and rapid phase is to accumulate osmolytes to maintain the turgor pressure [30, 31]. In the past decade, plant growth regulators have been extensively applied in researches to alleviate crop salinity stress including polyamines [32], epibrassinolide [33], jasmonate [34], melatonin [35], and silicon [36]; however, sustainable agricultural practices are still needed to achieve the goal of green ecological agriculture.

Being a leguminous crop, peanut (*Arachis hypogaea* L.) is a good source of protein and vegetable oil for humans [21, 37]. Peanut is relatively sensitive to saline stress [38–40]. Soil saline severely decreases seed germination, morphogenesis, and production of peanut [41–43]. In spite of this, peanut is often grown under poor soil conditions such as saline affected soil because nearly one-third of the global arable irrigated ploughland is already affected by salinity [44, 45]. As an important source of fiber, commercial cotton (*Gossypium hirsutum* L.) is moderately salt-tolerant [46, 47]. Recent years, peanut/cotton intercropping combined with rotation system has been increasingly adopted by farmers in North China Plain [13, 48] and elsewhere in the world [49]. The advantages of this cropping system could be to alleviate the constraints of continuous cropping and increase the productivity; however, whether this kind of intercropping system could be conducted under salinity conditions remains unknown. To address this concern, the current research was performed to elucidate the effects of peanut/cotton intercropping on crop productivity and economic returns under both normal and saline soil conditions. By using different root barriers, we were able to detect the interspecific facilitation between the below-ground parts of peanut strip and cotton strip. We therefore hypothesized that peanut/cotton intercropping could increase crop yield and economic returns through regulation of soil microbial communities and accumulation of nutrients in the crops. Our study may guide management decisions to enhance productivity in the era of soil salinization and develop sustainable agriculture.

## Methods

### Field experimental site

The multi-site field experiments were conducted at PingDu experimental station (120.12 °E, 36.55 °N), GaoTang experimental station (116.27 °E, 36.86 °N), and LiJin experimental station (118.27 °E, 37.50 °N), Shandong Province of China in 2018. All of the experimental sites are located in a warm semi-arid monsoon region with a continental climate during summer and autumn. No extreme weather conditions were observed during crop growth seasons in 2018. The temperature and rainfall were slightly different between these experimental sites from May to September in 2018. The soil was considered non-saline soil, secondary-saline soil, and coastal saline soil in PingDu, GaoTang, and LiJin, respectively which was classified according to the previous research [50]. The soil chemical properties of the top soil (0–20 cm and 20–40 cm) in each experimental site were listed in Table 1. Commercial cotton (var. Lumianyan 37) seeds obtained from Shandong Cotton Research Center and peanut (var. Huayu 25) seeds obtained from Shandong Peanut Research Institute were used in all of the experimental sites. Both peanut and cotton are major cultivated high-yield varieties in Shandong Province.

**Table 1:** Soil chemical properties of each experimental position

Position	Available N (mg kg^− 1^)		Available P (mg kg^− 1^)		Available K (mg kg^− 1^)		EC (mS cm^− 1^)		pH	
	0–20 cm	20–40 cm	0–20 cm	20–40 cm	0–20 cm	20–40 cm	0–20 cm	20–40 cm	0–20 cm	20–40 cm
PingDu	45.32	28.23	23.53	15.64	338.55	286.75	1.41	1.68	7.78	7.90
GaoTang	47.64	26.57	17.51	5.60	317.75	132.10	3.28	3.52	8.16	8.55
LiJin	48.82	22.34	22.97	4.90	129.45	93.65	4.07	4.31	8.01	8.40

### Experimental design

The managements were identical in all the 3 experimental sites. The fields were successively planted with peanuts, wheat, and maize for at least 10 consecutive years. Basal synthetic fertilizer (975 kg/hm^2^; N: P_2_O_5_: K_2_O = 1:1.5:1.5) was applied homogeneously into the soils before sowing. The peanut and cotton seeds with uniform sides were manually selected and sowed on 7 May, 23 April, and 3 May 2018 in PingDu, GaoTang, and LiJin, respectively.

**Fig. 1:**
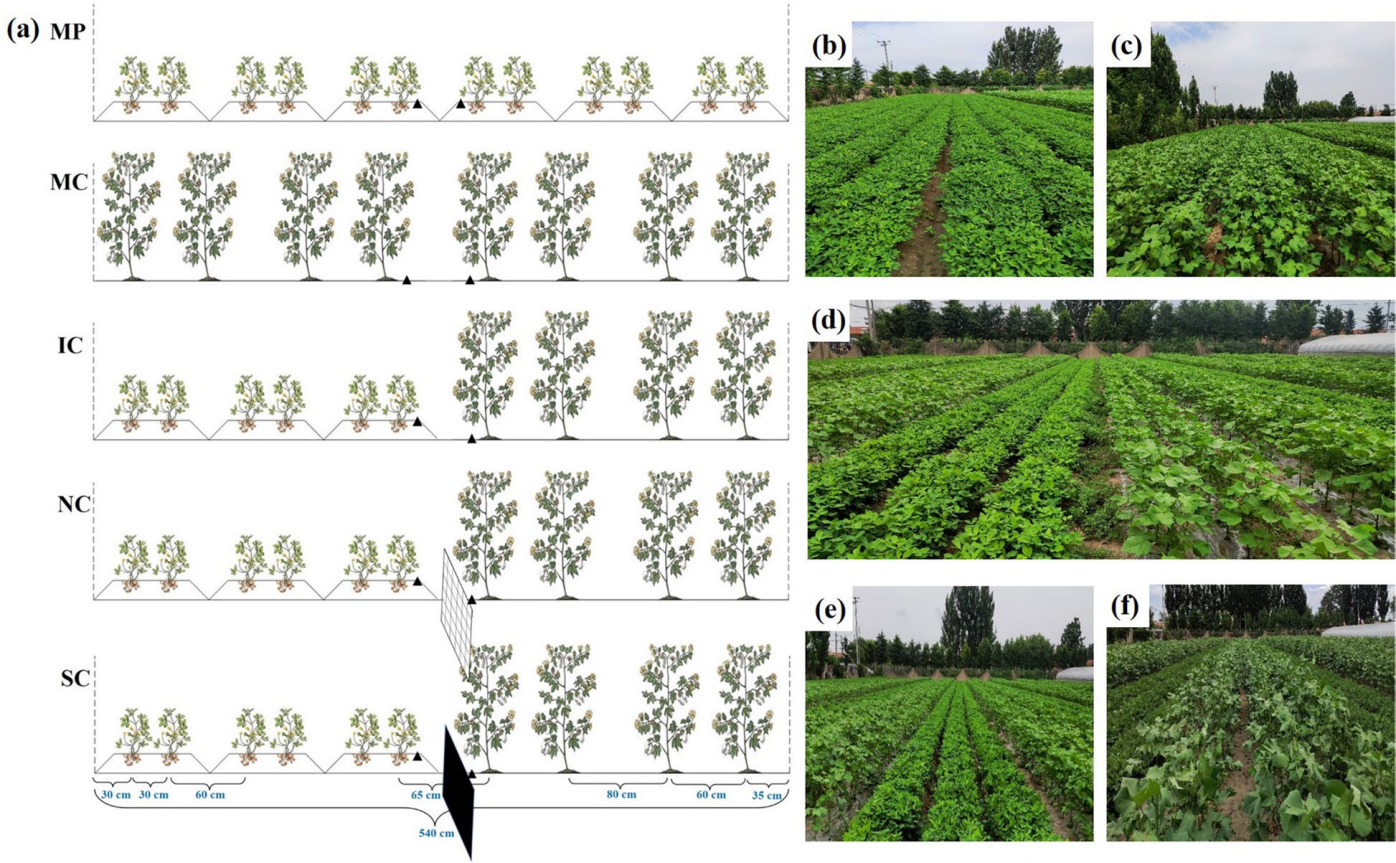
Schematic diagrams of experimental design used in different cropping systems and root barriers (**a**). MP: Monocropping of peanut, MC: Monocropping of cotton, IC: Intercropping of peanut/cotton without barriers, NC: Intercropping of peanut/cotton with 100 μm nylon mesh barrier, SC: Intercropping of peanut/cotton with solid barrier. Solid triangles represent soil sampling positions. Pictures showing MP (**b**), MC (**c**), both peanut and cotton in IC (**d**), peanut strips in IC (**e**), and cotton strips in IC (**f**)

The cropping system was modified as previously reported with minor modifications [13]. Peanut was sowed on a raised bed (Height 10 cm and width 80 cm). Two rows of peanut were arranged on a ridge (Row spacing 30 cm and plant distance 17 cm). Cotton was sowed in equal rows (Row spacing 60 cm and plant distance 25 cm). The spacing between cotton and peanut was 65 cm. There were 6 rows of peanut and 4 rows of cotton on each intercropping plot and the two crops occupied the same land area (270 cm in width) (Fig. 1). In order to eliminate the root interactions between peanut and cotton, two kinds of barriers were placed below-ground in the middle of the peanut strip and cotton strip. The permeable (100 μm nylon mesh) barrier was 15-m long and 1.5-m deep (No root contact whereas majority of root exudates and microbes can pass through) (NC) and the impermeable (solid plastic) barrier was 15-m long and 1.5-m deep (No below-ground contact) (SC). For no barrier intercropping treatment (IC), the ground between peanut strip and cotton strip has also been digged for 1.5-m deep and refilled as a negative control. The monocropping of peanut (MP) and cotton (MC) were identical with the intercropping system as shown in Fig. 1. Taken together, a total of five cropping systems were established, namely, monocropping of peanut (MP), monocropping of cotton (MC), intercropping of peanut/cotton without barriers (IC), intercropping of peanut/cotton with 100 μm nylon mesh barrier (NC), and intercropping of peanut/cotton with solid barrier (SC).

The experiments were set up using a randomized complete block design with three biological replicates in each treatment and the area of each plot was 162 m^2^ (15 m × 10.8 m). The peanut was manually harvested on 9 September, 2 September, and 22 September 2018 in PingDu, GaoTang, and LiJin, respectively while the cotton was manually harvested on 29 October, 20 October, and 22 October 2018 in PingDu, GaoTang, and LiJin, respectively.

### Measurements and data collection

#### Nutrient analyses from plant and soil samples

The plant and soil samples were taken simultaneously at peanut podding stage. Plant samples were firstly heated at 105 °C for 30 min to deactivate enzymes and dried at 75 °C to constant weight, and then the dry weights were immediately recorded. The samples were finely ground to powder and digested with H_2_SO_4_-H_2_O_2_ as separate plant parts. Nitrogen (N) content was assayed using the micro Kjeldahl analysis [51]. Phosphorus (P) content was determined by a flow analyzer according to the manufacturer’s instructions [52]. Potassium (K) content was measured using a flame photometer as described by K Chakraborty, D Bhaduri, HN Meena and K Kalariya [53].

Freshly collected soil samples of the top soil (0–40 cm) which were taken at 15 cm away from the plants in the peanut strip and cotton strip (as shown in Fig. 1) were sieved to 2 mm and the identical methodology was followed except the extraction to determine the content of soil N, P, and K.

#### Yield and yield components

Yield samples were taken at 1 day before harvest. After sun-drying for 15 days, the yield and yield components of peanut and cotton were measured, respectively. The sampling area for both sole peanut and sole cotton was 13.5 m^2^ (5 m in length and 2.7 m in width). For peanut, the pods yield, pod density, and 100-pod weight were measured for all plants in sampling area. For cotton, all plants were collected in sampling area to quantify the seed cotton yield, boll density, and boll weight.

#### Competition parameters

Land equivalent ratio (LER) was utilized to evaluate the land use advantage provided by intercropping [54]. LER was calculated as:

$$\mathrm{LER}=\mathrm{LERp}+\mathrm{LERc}= Yp/ Mp+ Yc/ Mc$$

where LERp and LERc are partial LERs for peanut and cotton, respectively, *Yp* and *Mp* are the yields of peanut in intercropping and monocropping systems, respectively, and *Yc* and *Mc* are the yields of cotton in intercropping and monocropping systems, respectively. Ratios of 1.0 indicate the same land productivity for intercropping and monocropping systems, ratios greater than 1.0 indicate that intercropping is advantageous, whereas ratios smaller than 1.0 indicate a land use disadvantage for intercropping.

#### Benefit-cost measurement

Material inputs such as seeds, fertilizer, pesticide, irrigation system, and labor cost including fertilizer application, irrigation, insect and weed control, and harvesting were recorded in each experimental station. The input cost was calculated based on the local prices of the material and labor days, meanwhile, the output cost of the peanut pods and seed cotton was determined according to the average prices of the local market in 2018.

#### Soil sampling and determination of soil bacterial communities

Soil samples were taken from both 0–20 cm and 20–40 cm depth on 15 July (blossom-needling stage of peanut) and 25 August (podding stage of peanut) in PingDu. The sampling positions were 15 cm away from the plants in the peanut strip and cotton strip as marked in solid triangles (Fig. 1). Soil samples were collected from 3 random points within each replicate plot and mixed thoroughly, and each treatment composed of 3 replicates. In MP, MC, and IC, soil samples from the two strips were further mixed together to make the composite samples. In NC and SC, the peanut strip and cotton strip samples were collected separately as NC-P, NC-C, SC-P, and SC-C, respectively. The obtained soil samples were firstly frozen in liquid nitrogen and then immediately transported to Gene Denovo Biotechnology Co., Ltd., Guangzhou, China in drikold for DNA extraction and soil microbial determinations.

High-throughput sequencing analysis of the 16S rRNA gene was performed with Illumina Novaseq 6000 to determine soil bacterial diversities and communities as previously described [52]. Total genomic DNA was extracted from 0.5 g soil using the HiPure Soil DNA Kit (Magen, Guangzhou, China) following the manufacturer’s protocols [55]. The hypervariable regions of V3 and V4 of the 16S rRNA genes were amplified using the specific primers 341F (5′-CCT ACG GGN GGC WGC AG-3′) and 806R (5′-GGA CTA CHV GGG TAT CTA AT-3′) according to A Ali, M Imran Ghani, Y Li, H Ding, H Meng and Z Cheng [56]. The concentration and quality of the DNA samples were firstly determined before polymerase chain reaction (PCR) amplification was carried out. Amplicons extracted from 2% agarose gels were further purified with a AxyPrep DNA Gel Extraction Kit (Axygen Biosciences, Union City, CA, USA). The purified triplicate amplification products were pooled in equimolar amounts and quantified using NanoDrop (Thermo Scientific, USA) according to standard protocols [42]. Afterwards, the prepared libraries were sequenced on an Illumina HiSeq 2500 PE 250 platform. The raw sequences data generated in the current research were deposited into the National Center for Biotechnology Information (NCBI) Sequence Read Archive (SRA) database under Submission ID of SUB10633613 and Accession of PRJNA779843.

Raw data containing adapters or low quality reads would affect the following assembly and analysis. To get high quality clean reads, raw reads were further filtered when they were containing more than 10% of unknown nucleotides or containing less than 80% of bases with quality (Q-value) > 20. Consequently, effective tags were used for the subsequent analysis. Then, paired end clean reads were merged as raw tags using FLSAH (V1.2.11) according to T Magoč and SL Salzberg [57] with a minimum overlap of 10 bp and mismatch error rates of 2%. Noisy sequences of raw tags were filtered by QIIME (V1.9.1) [58] pipeline under specific filtering conditions [59] to obtain the high quality clean tags. Then, clean tags were searched against the reference database (http://drive5.com/uchime/uchime_download.html) to perform Reference based chimera checking using UCHIME algorithm (http://www.drive5.com/usearch/manual/uchime_algo.html). All chimeric tags were removed and finally obtained effective tags were used for further analysis.

The effective tags were clustered into operational taxonomic units OTUs of ≥97% similarity using UPARSE [60] pipeline. The tag sequence with highest abundance was selected as reprehensive sequence within each cluster. The representative sequences were classified into organisms by a naive Bayesian model using RDP classifier (V2.2) [61] based on UNITE Database (https://unite.ut.ee/). The abundance statistics of each taxonomy and phylogenetic tree was construction in a Perl script and visualized using SVG [62]. Biomarker features in each group were screened by Metastats and LEfSe software. Additionally, Chao1, Simpson, and all other alpha diversity index were calculation in QIIME. OTU rarefaction curve and Rank abundance curves was plotted in QIIME. Alpha index comparing among groups was computed by a Tukey’s HSD test and a Kruskal-Wallis H test in R. The principal coordinates analysis (PCoA) in Hellinger distance was calculated and plotted in R. The metabolic functional features of the bacterial communities were predicted using Tax4Fun (version 1.0) [63] with Kyoto Encyclopedia of Genes and Genomes (KEGG) database [64].

### Statistical analysis

The physiological data were firstly tested for homogeneity of variance with boxplot and subjected to the one-way analysis of variance (ANOVA). The difference was considered to be statistically significant when *P* < 0.05 using Tukey’s test.

## Results

### Yield and yield components of peanut and cotton

The highest peanut pods yield was observed in treatment MP (5192 kg/ha in PingDu, 4967 kg/ha in GaoTang, and 5146 kg/ha in LiJin), while the lowest peanut pods yield was obtained under treatment SC (3816 kg/ha in PingDu, 3775 kg/ha in GaoTang, and 3726 kg/ha in LiJin) (Table 2). In addition, the maximum seed cotton yield was found under IC treatment (7160 kg/ha in PingDu, 6846 kg/ha in GaoTang, and 6778 kg/ha in LiJin), whereas the lowest seed cotton yield (4420 kg/ha in PingDu, 4124 kg/ha in GaoTang, and 4276 kg/ha in LiJin) was recorded in MC treatment in 3 positions (Table 3). Intercropping with root barriers significantly reduced peanut pods yield by 9.34% (NC) and 14.54% (SC) in PingDu, 11.10% (NC) and 15.55% (SC) in GaoTang, and 8.43% (NC) and 13.27% (SC) in LiJin, compared with no root barrier treatment (IC) (Table 2). In seed cotton yield, the reduction was 17.26% (NC) and 20.67% (SC) in PingDu, 12.05% (NC) and 16.87% (SC) in GaoTang, and 10.70% (NC) and 14.46% (SC) in LiJin, compared with IC (Table 3).

**Table 2:** Yield and yield components of peanut in different cropping systems

Position	Treatment	Peanut pods yield (kg ha^−1^)	Yield components	
			Pod density (pods m^−2^)	100-pod weight (g)
PingDu	MP	5192a	190.9a	272a
	IC	4465b	163.0b	274a
	NC	4048c	152.2c	266b
	SC	3816d	144.9 cd	263b
GaoTang	MP	4967a	189.3b	262a
	IC	4470b	224.9a	199bc
	NC	3974c	182.7b	217b
	SC	3775c	182.5b	207b
LiJin	MP	5146a	196.7a	262a
	IC	4296b	161.7b	266a
	NC	3934c	151.9c	259ab
	SC	3726c	147.3c	253b

Means denoted by different letters within the same column of the same position indicate significant differences according to Tukey’s test (*P* < 0.05); MP: monocropping of peanut; IC: intercropping of peanut and cotton without barriers; NC: intercropping of peanut/cotton with 100 μm nylon mesh barrier; SC: intercropping of peanut/cotton with solid barrier

**Table 3:** Seed cotton yield and yield components in different cropping systems

Position	Treatment	Seed cotton yield (kg ha^− 1^)	Yield components	
			Boll density (bolls m^−2^)	Boll weight (g boll^− 1^)
PingDu	MC	4420d	82.53d	5.36a
	IC	7160a	158.18a	4.53b
	NC	5924b	133.78b	4.43b
	SC	5680c	105.86c	5.37a
GaoTang	MC	4124d	80.69c	5.11bc
	IC	6846a	115.93a	5.91a
	NC	6021b	117.23a	5.14b
	SC	5691c	108.83b	5.23b
LiJin	MC	4276d	73.39d	5.83b
	IC	6778a	108.66b	6.24a
	NC	6053b	114.20a	5.30c
	SC	5798c	98.83c	5.87b

Means denoted by different letters within the same column of the same position indicate significant differences according to Tukey’s test (*P* < 0.05); MC: monocropping of cotton; IC: intercropping of peanut and cotton without barriers; NC: intercropping of peanut/cotton with 100 μm nylon mesh barrier; SC: intercropping of peanut/cotton with solid barrier

We then measured the peanut pod density and cotton boll density where intercropping (IC) significantly decreased peanut pod density by 14.61 and 17.79% in PingDu and LiJin, respectively, while increased peanut pod density by 18.81% in GaoTang compared with MP. The boll density of cotton in IC was significantly increased by 91.66, 43.67, and 48.06% in PingDu, GaoTang, and LiJin, respectively compared with MC. Root barriers significantly reduced the pod density of peanut and the reduction was 6.63% (NC) and 11.10% (SC) in PingDu, 18.76% (NC) and 18.85% (SC) in GaoTang, and 6.06% (NC) and 8.91% (SC) in LiJin compared with IC. The changes of boll density of cotton by root barriers were − 15.43% (NC) and − 33.08% (SC) in PingDu, 1.12% (NC) and − 6.12% (SC) in GaoTang, and 5.10% (NC) and − 9.05% (SC) in LiJin, compared with IC. Intercropping did not change 100-pod weight of peanut except for that in GaoTang where 100-pod weight was significantly decreased by 24.05% compared with MP. Intercropping significantly reduced boll weight of cotton by 15.49% in PingDu, while increased boll weight of cotton by 15.66 and 7.03% in GaoTang and LiJin, respectively, compared with IC. Compared with IC, the 100-pod weight was reduced (2.92% of NC and 4.01% of SC) in PingDu and (2.63% of NC and 4.89% of SC) in LiJin, while induced (9.05% of NC and 4.02% of SC) in GaoTang. In PingDu, SC significantly increased boll weight of cotton by 18.54% while NC did not change this parameter, compared with IC. In GaoTang and LiJin, root barriers significantly reduced boll weight of cotton by (13.03% of NC and 11.51% of SC) and (15.06% of NC and 5.93% of SC), respectively, compared with IC (Tables 2 and 3).

### Competition parameters

In general, the value of LER for all of the treatments were found higher than one suggesting yield advantage of peanut/cotton intercropping system. Among all the treatments, IC had the maximum LER: 1.24, 1.28, and 1.21 in PingDu, GaoTang, and LiJin, respectively, while lowest LER was recorded by 1.01, 10.7, and 1.04 in PingDu, GaoTang, and LiJin, respectively, under treatment SC. Relative to NC, treatment SC significantly reduced LER by 4.72, 5.31, and 4.59% in PingDu, GaoTang, and LiJin, respectively. Similar changes of LERp and LERc were further observed where root barriers significantly reduced LERp and LERc in all of the experimental positions except that LERp did not show significant differences between SC and NC in 3 positions (Table 4).

**Table 4:** LER for peanut and cotton in different root barrier treatments and positions

Treatment	PingDu			GaoTang			LiJin		
	LERp	LERc	LER	LERp	LERc	LER	LERp	LERc	LER
IC	0.43a	0.81a	1.24a	0.45a	0.83a	1.28a	0.42a	0.79a	1.21a
NC	0.39b	0.67b	1.06b	0.40b	0.73b	1.13b	0.38b	0.71b	1.09b
SC	0.37b	0.64c	1.01c	0.38b	0.69c	1.07c	0.36b	0.68c	1.04c

Means denoted by different letters within the same column indicate significant differences according to Tukey’s test (*P* < 0.05); IC: intercropping of peanut and cotton without barriers; NC: intercropping of peanut/cotton with 100 μm nylon mesh barrier; SC: intercropping of peanut/cotton with solid barrier; LERp denotes partial LER for peanut; LERc denotes partial LER for cotton

### Economic analysis

Input value, output value, and net return were significantly affected by the cropping systems. Overall, the cost of inputs for IC was between MP and MC in all experimental positions. IC produced a significantly higher output value (24.21% of MP and 4.24% of MC) in PingDu, (22.49% of MP and 5.38% of MC) in GaoTang, and (22.92% of MP and 5.38% of MC) in LiJin. Averaged for 3 positions, IC produced a significantly higher output value by 23.76 and 4.98% in MP and MC, respectively. Compared with MP, the net returns in IC were significantly increased by 20.08, 10.90, and 15.22% in PingDu, GaoTang, and LiJin, respectively. The net returns in IC were significantly increased by 54.95, 67.31, and 65.78% in PingDu, GaoTang, and LiJin, respectively, compared with MC. Averaged for 3 positions, the net returns in IC were significantly increased by 15.47 and 62.18% compared with MP and MC, respectively (Table 5).

**Table 5:** Output, input and net return as affected by intercropping systems

Position	Pattern	Input ($ ha^−1^)	Output ($ ha^−1^)	Return ($ ha^−1^)
PingDu	MP	2215c	3779c	1564b
	MC	3291a	4503b	1212c
	IC	2816b	4694a	1878a
GaoTang	MP	2071c	3615c	1495b
	MC	3210a	4202b	991c
	IC	2770b	4428a	1658a
LiJin	MP	2166c	3744c	1577b
	MC	3271a	4367b	1096c
	IC	2785b	4602a	1817a
Average	MP	2151c	3696c	1545b
	MC	3257a	4357b	1100c
	IC	2790b	4574a	1784a

Means denoted by different letters within the same column of the same position indicate significant differences according to Tukey’s test (*P* < 0.05); MP: monocropping of peanut; MC: monocropping of cotton; IC: intercropping of peanut and cotton; Peanut seeds: 5.0 ¥ kg^−1^, cotton seeds: 7.0 ¥ kg^−1^; Labor cost for peanut: 8400 ¥ ha^− 1^ (80 ¥ man-days^− 1^ × 105 man-days ha^− 1^); Labor cost for cotton: 15200 ¥ ha^− 1^ (80 ¥ man-days^− 1^ × 190 man-days ha^− 1^); Material input for peanut: 6517 ¥ ha^− 1^ in PingDu, 6430 ¥ ha^− 1^ in GaoTang, and 6483 ¥ ha^− 1^ in LiJin; Material input for cotton: 7410 ¥ ha^− 1^ in PingDu, 6855 ¥ ha^− 1^ in GaoTang and 7270 ¥ ha^− 1^ in LiJin. Exchange rate: 6.87 ¥ ≈ 1US $

### Nutrient accumulation in soil and plants of peanut and cotton

The N, P, and K accumulation in the soil of peanut and cotton strips were significantly greater in IC than MP or MC in all 3 experimental positions with only one exception where IC decreased the peanut strip K by 9.53% in GaoTang (Table 6). However, for NC treatment, the N, P, and K accumulation in the soil of peanut and cotton strips were significantly lower than IC in all 3 experimental positions with 3 exceptions where N accumulation of peanut strip was increased by 2.77% in PingDu, P accumulation of cotton strip was increased by 6.71% in GaoTang, and K accumulation of peanut strip was increased by 25.50% in GaoTang. In addition, the significantly lower accumulation of N, P, and K in the soil of peanut and cotton strips of SC treatment was recorded compared with IC, suggesting that solid root barriers might inhibited the accumulation of the soil nutrient in both peanut and cotton strips (Table 6).

**Table 6:** Contents of soil N, P, and K in peanut and cotton strips of different cropping systems

Position	Treatment	Peanut strip N (mg kg^− 1^)	Cotton strip N (mg kg^− 1^)	Peanut strip P (mg kg^− 1^)	Cotton strip P (mg kg^− 1^)	Peanut strip K (mg kg^− 1^)	Cotton strip K (mg kg^− 1^)
PingDu	MP	40.16c	–	15.27b	–	250.16d	–
	MC	–	43.78c	–	16.73b	–	201.98c
	IC	50.24b	51.63a	19.66a	19.66a	324.15a	300.11a
	NC	51.63a	46.81b	18.56a	17.60b	283.35b	181.73d
	SC	35.46d	34.51d	13.24c	13.52c	261.74c	283.17b
GaoTang	MP	45.63c	–	18.94b	–	445.97b	–
	MC	–	52.41c	–	15.10c	–	330.00d
	IC	53.11a	57.86a	21.97a	19.97b	403.45c	432.71a
	NC	47.35b	56.43b	17.24c	21.31a	506.31a	425.31b
	SC	43.44d	43.14d	15.23d	15.02c	253.47d	345.14c
LiJin	MP	25.02c	–	16.54c	–	323.67d	–
	MC	–	25.37b	–	11.86c	–	184.97d
	IC	31.23a	31.44a	29.41a	29.41a	473.65a	473.91a
	NC	29.61b	30.69a	24.96b	18.83b	438.68b	211.12c
	SC	25.71bc	21.31c	16.15c	11.15c	381.41c	351.43b

Means denoted by different letters within the same column of the same position indicate significant differences according to Tukey’s test (*P* < 0.05); MP: monocropping of peanut; MC: monocropping of cotton; IC: intercropping of peanut and cotton without barriers; NC: intercropping of peanut/cotton with 100 μm nylon mesh barrier; SC: intercropping of peanut/cotton with solid barrier

The accumulation of N in the stem and leaf of peanut did not show significant difference between MP and IC, while IC significantly changed cotton N in the stem and leaf of PingDu and GaoTang compared with MC (Table 7). For the accumulation of N in peanut pod, IC showed significantly higher N accumulation than MP in 3 positions and higher accumulation of N in cotton bud was also found in IC compared with MC in PingDu and GaoTang. Root barrier treatment NC significantly increased the accumulation of N in the stem and leaf of both peanut and cotton compared with IC in GaoTang and LiJin while the changes were not unique in peanut pod and cotton bud when compared between NC and IC. SC significantly reduced the accumulation of peanut pod N in 3 positions and cotton bud N in PingDu and LiJin, compared with IC.

**Table 7:** Contents of N, P, and K of various organs in different cropping systems

Position	Organ	Treatment	N		P		K	
			Peanut N (mg kg^−1^)	Cotton N (mg kg^−1^)	Peanut P (mg kg^−1^)	Cotton P (mg kg^−1^)	Peanut K (mg kg^− 1^)	Cotton K (mg kg^− 1^)
PingDu	Stem+Leaf	MP	44.70a	–	41.87b	–	228.02c	–
		MC	–	46.21b	–	54.43b	–	238.39d
		IC	43.61a	49.03a	41.23b	57.32a	260.59b	274.00a
		NC	43.52a	49.87a	44.75a	56.54a	274.97a	267.30b
		SC	43.71a	46.21b	40.70b	56.65a	214.69d	251.97c
	Pod/Bud	MP	32.12c	–	44.44b	–	147.57c	–
		MC	–	39.09b	–	85.31d	–	201.54d
		IC	47.9a	44.64a	60.36a	106.27a	178.05a	228.56a
		NC	36.4b	45.07a	45.34b	104..64b	165.09b	205.65c
		SC	32.2c	43.03c	39.37c	101.47c	143.04d	207.32b
GaoTang	Stem+Leaf	MP	30.91ab	–	41.17b	–	279.05c	–
		MC	–	37.31a	–	37.09b	–	233.58d
		IC	30.26b	35.46b	49.61a	38.16ab	284.50b	253.91b
		NC	31.61a	36.81a	49.08a	39.23a	288.97a	251.47c
		SC	29.93b	36.43b	37.91c	37.80b	271.64d	270.70a
	Pod/Bud	MP	42.44b	–	40.96c	–	66.81c	–
		MC	–	38.96c	–	39.14c	–	134.82c
		IC	46.47a	40.12b	43.04b	43.60a	72.45a	167.41a
		NC	47.87a	41.78a	44.50a	42.35b	68.73b	139.83b
		SC	41.13c	41.33a	33.06d	39.71c	65.46d	133.12d
LiJin	Stem+Leaf	MP	35.53b	–	50.28b	–	223.36c	–
		MC	–	42.81b	–	52.53c	–	249.87d
		IC	35.41b	42.93b	52.52a	56.60a	228.07b	342.51b
		NC	36.88a	45.96a	51.19b	56.76a	235.87a	353.30a
		SC	33.63c	38.71c	48.61c	55.27b	220.91d	309.16c
	Pod/Bud	MP	38.30b	–	39.67b	–	100.38c	–
		MC	–	45.13a	–	49.27d	–	162.13d
		IC	39.61a	45.16a	43.09a	61.70a	103.87a	259.40b
		NC	40.72a	33.45c	43.62a	55.02b	102.31b	268.68a
		SC	38.27b	42.62b	37.38c	51.34c	99.46c	257.25c

Means denoted by different letters within the same column of the same position indicate significant differences according to Tukey’s test (*P* < 0.05); MP: monocropping of peanut; MC: monocropping of cotton; IC: intercropping of peanut and cotton without barriers; NC: intercropping of peanut/cotton with 100 μm nylon mesh barrier; SC: intercropping of peanut/cotton with solid barrier

IC significantly increased the accumulation of peanut P in the stem and leaf of GaoTang and LiJin compared with MP and increased the cotton P in the stem and leaf of PingDu and LiJin compared with MC. Additionally, IC showed significantly higher P accumulation in peanut pod and cotton bud of 3 positions, compared with MP and MC. Root barrier treatment NC and SC showed lower P accumulation in the stem, leaf, peanut pod, and cotton bud of the seedlings in 3 positions than IC with a few exceptions, suggesting that root barriers inhibited the accumulation of P in both peanut and cotton plants.

For the K accumulation, intercropping increased the K in the stem, leaf, peanut pod, and cotton bud within a certain range compared with MP and MC. In peanut, NC showed significant higher K in stem and leaf while lower K in the pod, compared with IC. SC showed significant lower K in all of the organs of peanut compared with IC. In cotton, similar results were observed where root barrier treatment NC and SC significantly reduced the K in all of the organs in 3 positions except the SC of stem and leaf in GaoTang and NC of all of the organs in LiJin, compared with IC. These results indicated that root barriers reduced the accumulation of plant K in the organs of both cotton and peanut (Table 7).

### Soil bacterial communities

Most of the root barrier intercropping treatments significantly affected diversity indices and affected community structure of soil bacterial community in both peanut strip and cotton strip (Table S1). At blossom-needling stage (0–20 cm), MP showed significantly higher number of OTUs, ACE, and Chao index values than other treatments, while at blossom-needling stage (20–40 cm), NC-C showed significantly higher number of OTUs, Shannon index, ACE, and Chao index values compared with other treatments. Again, NC-C processed higher number of OTUs, ACE, and Chao index values than other treatments, whereas SC-C processed the highest Shannon and Simpson index values at podding stage (0–20 cm). In 20–40 cm top soil of podding stage, however, only NC-C showed significant higher Shannon index values compared with other treatments (Table S1).

**Fig. 2:**
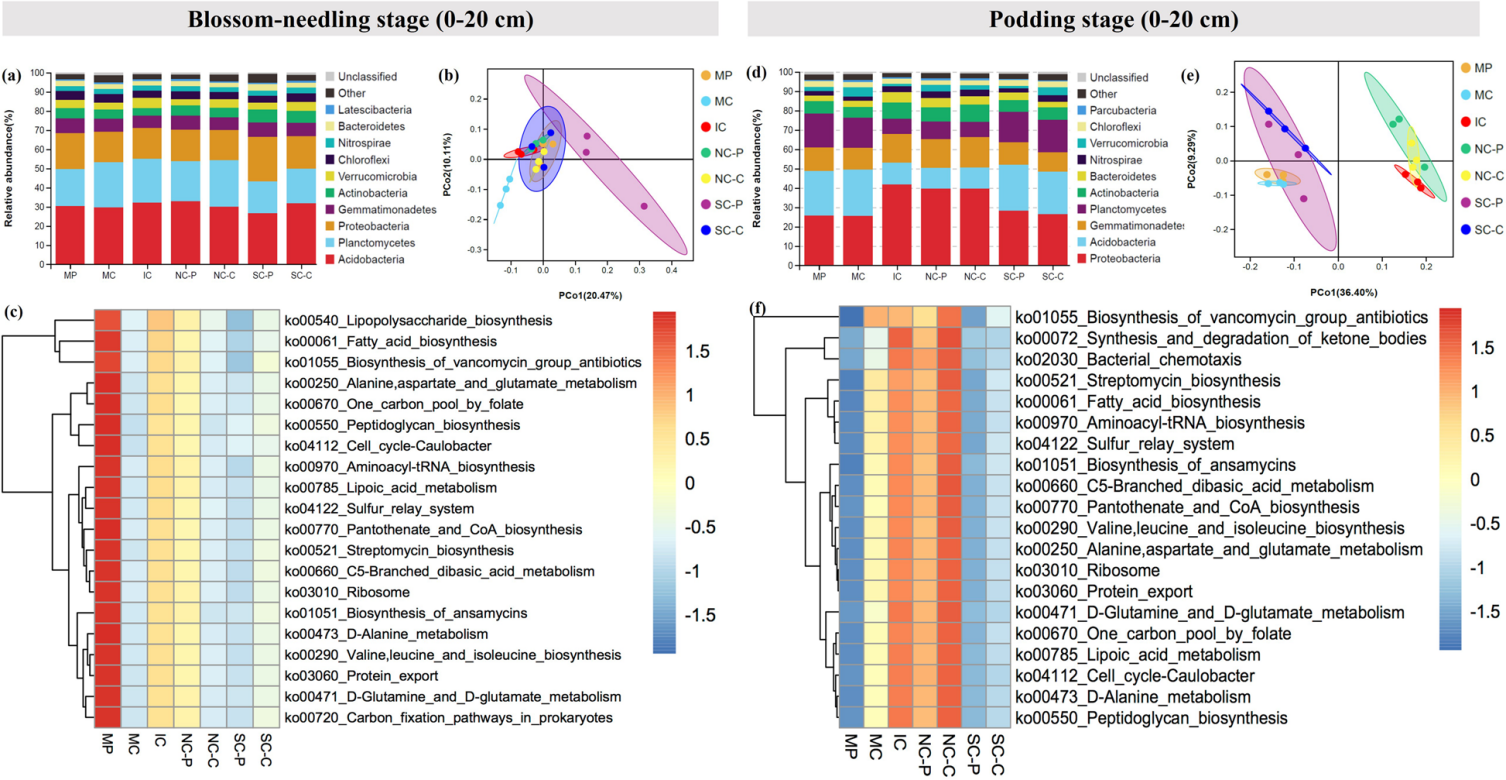
The relative abundance of soil bacterial proportions (0–20 cm) at the phylum level at blossom-needling stage (**a**) and podding stage (**d**). The principal coordination analysis (PCoA) in Hellinger distance at blossom-needling stage (**b**) and podding stage (**e**) showing changings in soil bacterial community structure. Heatmap based most abundant Kyoto Encyclopedia of Genes and Genomes (KEGG) ortholog (KO) groups showing the relative abundance of top 20 KEGG metabolic pathways across different treatments at blossom-needling stage (**c**) and podding stage (**f**) in monocropped peanut (MP), monocropped cotton (MC), peanut intercropped with cotton without barriers (IC), peanut intercropped with cotton with 100 μm nylon mesh barrier: peanut strip (NP) and cotton strip (NC), peanut intercropped with cotton with solid barrier: peanut strip (SP) and cotton strip (SC). The samples were taken from 0 to 20 cm of the top soil

**Fig. 3:**
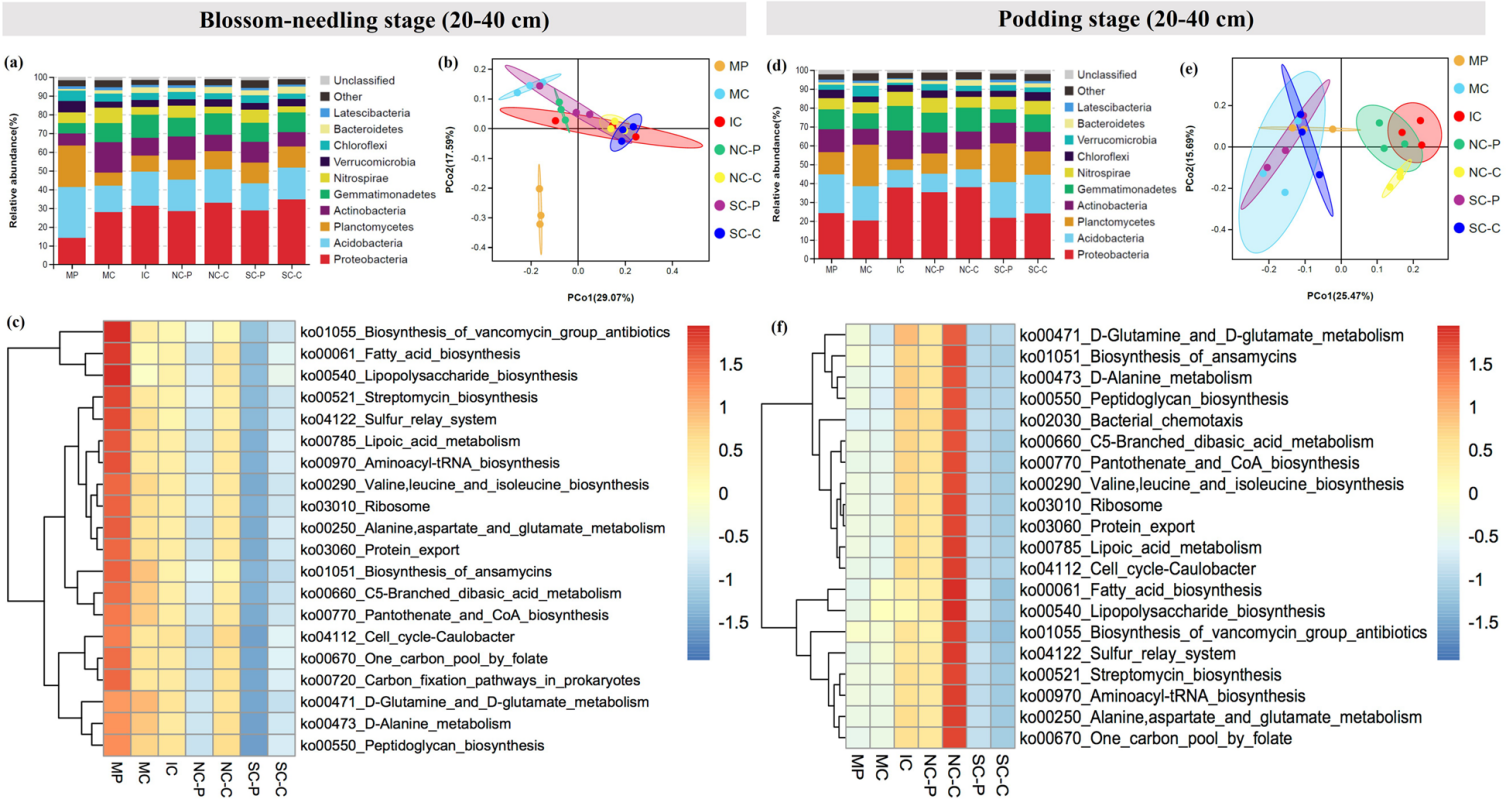
The relative abundance of soil bacterial proportions (20–40 cm) at the phylum level at blossom-needling stage (**a**) and podding stage (**d**). The principal coordination analysis (PCoA) in Hellinger distance at blossom-needling stage (**b**) and podding stage (**e**) showing changings in soil bacterial community structure. Heatmap based most abundant Kyoto Encyclopedia of Genes and Genomes (KEGG) ortholog (KO) groups showing the relative abundance of top 20 KEGG metabolic pathways across different treatments at blossom-needling stage (**c**) and podding stage (**f**) in monocropped peanut (MP), monocropped cotton (MC), peanut intercropped with cotton without barriers (IC), peanut intercropped with cotton with 100 μm nylon mesh barrier: peanut strip (NP) and cotton strip (NC), peanut intercropped with cotton with solid barrier: peanut strip (SP) and cotton strip (SC). The samples were taken from 20 to 40 cm of the top soil

At the phylum level, IC obviously raised the relative abundance of Acidobacteria and Verrucomicrobia, while declined the relative abundance of Gemmatimonadetes, Actinobacteria, and Chloroflexi compared with MP and MC at blossom-needling stage (0–20 cm). NC-P and NC-C did not show obvious changes of the bacterial communities, whereas SC-P and SC-C visibly increased the relative abundance of Proteobacteria and Actinobacteria, and greatly declined the relative abundance of Planctomycetes, and Verrucomicrobia compared with IC (Fig. 2a & Fig. S1). At podding stage (0–20 cm), IC greatly induced the relative abundance of Proteobacteria, Gemmatimonadetes, Actinobacteria, and Bacteroidetes whereas reduced the relative abundance of Acidobacteria, Planctomycetes, and Verrucomicrobia compared with MP and MC. NC-P and NC-C did not show significant changes compared with IC. By contrast, the relative abundance of Proteobacteria, Gemmatimonadetes, Actinobacteria, and Bacteroidetes were obviously declined while the relative abundance of Acidobacteria and Planctomycetes were clearly elevated in SC-P and SC-C compared with IC (Fig. 2d). At blossom-needling stage (0–20 cm), the relative abundance of Proteobacteria, Gemmatimonadetes, and Bacteroidetes were induced while the relative abundance of Nitrospirae and Chloroflexi were reduced in IC compared with MP and MC. Compared NC-P and SC-P with IC, the relative abundance of Planctomycetes, Actinobacteria, and Nitrospirae were increased while Proteobacteria, Gemmatimonadetes, and Bacteroidetes were decreased. Compared NC-C and SC-C with IC, the relative abundance of Proteobacteria and Planctomycetes were induced while Actinobacteria, and Gemmatimonadetes were reduced (Fig. 3a & Fig. S1). At blossom-needling stage (20–40 cm), the relative abundance of Proteobacteria, Actinobacteria, Gemmatimonadetes, Nitrospirae, and Bacteroidetes were higher while Acidobacteria, Planctomycetes, Verrucomicrobia, and Latescibacteria were lower in IC than MP and MC. Additionally, the relative abundance of Planctomycetes and Verrucomicrobia showed higher whereas Actinobacteria, and Gemmatimonadetes showed lower in NC-P and NC-C than IC. SC-P and SC-C processed higher relative abundance of Acidobacteria, Planctomycetes, Verrucomicrobia, and Latescibacteria while lower Proteobacteria, Actinobacteria, Gemmatimonadetes, and Nitrospirae compared with IC (Fig. 3d).

The PCoA analysis revealed evident change in soil community structure of different cropping systems and the bacterial community of all of the treatments grouped well. At blossom-needling stage, no obvious difference was detected among treatments (Figs. 2b & 3b). However, IC, NC-P, and NC-C exerted a distinct difference as compared to the communities of other treatments in both 0–20 and 20–40 cm of the top soil at podding stage (Figs. 2e & 3e).

We then used the novel Tax4Fun tool to further explain the predictive functional profiling of microbial communities. At blossom-needling stage, the metabolic functions related to fatty acid biosynthesis, lipoic acid metabolism, peptidoglycan biosynthesis, biosynthesis of ansamycins, D-Alanine metabolism, cell cycle-Caulobacter, sulfur rely system, ribosome, protein export, etc. were significantly higher in the MP group in both 0–20 and 20–40 cm of the top soil especially compared with SC-P and SC-C groups (Figs. 2c & 3c). Strikingly, these putative KEGG pathways were significantly depleted in MP group, but visibly enriched in IC, NC-P, and NC-C groups at podding stage in both 0–20 and 20–40 cm of the top soil (Figs. 2f & 3f). These metabolic results indicate differential regulation of the soil bacterial community functional profiles by the different cropping systems and crop growth stages.

## Discussion

The main objective of this paper was to present a peanut/cotton intercropping system that significantly raised the crop productivity and economic returns under non-saline, secondary-saline, and coastal saline soil conditions. The present intercropping system also showed significant advantages over traditional monoculture systems. Based on multi-site field experiments with permeable (100 μm) and impermeable (solid) root barriers, we demonstrated that this phenomenon could be mainly attributed to the altering of the soil bacterial abundance distribution which can enhance the nutrient accumulation including N, P, and K in the soils, peanut pods, and cotton buds. Similarly, Chi et al. examined the advantages of this intercropping system from the point of leaf photosynthesis, dry matter partitioning, and the interactions between border and inner rows [13]; however, our study provides evidence from the perspective of the below-ground interspecific interactions and clear indicate the involvement of soil microbial community in peanut and cotton interaction. These findings highlight the importance of this promising intercropping system and provide an important reference for farmers to lower inputs and obtain more economic returns under various soil conditions.

In the peanut/cotton intercropping system, cotton is a tall crop with a high ground cover from June to September which is in a dominant position; however, peanut is a short crop during the whole co-growth phase except seedling stage which is in a disadvantage position [14]. This could be ascribed to the strong competition of cotton such as light, nutrients, and water [13, 65]. In conformity with earlier reports, we observed that intercropping clearly decreased the peanut pods yield while profoundly increased seed cotton yield compared with their monocultures [13, 49]. Strikingly, the reduction of peanut pods yield were even more aggravated under root barrier treatments especially in solid barrier treatment (SC). Similar results were obtained in seed cotton yield where NC and SC partially eliminated the beneficial effects of intercropping on cotton productivity (Tables 2 & 3). These results have been further demonstrated by land use efficiency of intercropping as quantified by LER where NC and SC dramatically decreased the LER compared with IC (Table 4). Therefore, we speculate that the above observations could be mainly ascribed to the below-ground interactions between the two crop species.

It is well accepted that nutrient translocation from soil to below-ground and from below-ground to above-ground components of crops could further influence the concentrations of these nutrients in peanut pods and cotton buds [66–68]. Consistent with previous reports, we observed that intercropping accelerated the accumulations of N, P, and K at various degrees in both peanut and cotton strips of soil compared with their monocultures (Table 6). It can be presumed that this intercropping system could promote the adequate availability and improve the acquisition of these nutrients in soil [4]. Indeed, crops are known to benefit from intercropping with legumes such as peanut for the symbiotic relationship between rhizobia and legume roots could provide more available N for their neighboring crop species [69–71]. It is worth noting that the enhanced accumulation of N was only observed in stems, leaves, and buds of cotton of IC treatment; however, although intercropping stimulated the uptake of N in the above-ground parts of peanut, it failed to increase this nutrient’s concentration in peanut pods (Table 7). These discrepancies might result from the reduced allocation of N from the vegetative tissues to the reproductive organs which warrant further investigation. By contrast, the accumulation of P and K in vegetative tissues of peanut and cotton showed similar trends as those in the soil where IC dramatically increased these nutrient contents compared with their monocultures (Table 7). Particularly, the induction was more pronounced in cotton than peanut plants while the reduction by SC was more pronounced than NC. On the one hand, this phenomenon might be due to the competition effects of the above-ground parts where higher light interception and leaf transpiration at the top of cotton than peanut could enhance the absorption capacity of nutrients [11, 72]. On the other hand, the interactions of the below-ground part of the two species prompted us to further elucidate the underlying mechanisms.

It has been well documented that high biodiversity increases ecosystem functions [15, 56, 73]. Unexpectedly, limited changes of soil bacterial community diversity and richness in the peanut/cotton intercropping system were detected compared with their monocultures (Table S1). By contrast, the bacterial community composition of soil changed dramatically in various treatments (Figs. 2 & 3). The discrepancies between the previous researches and ours might resulted from the two major reasons. First, rhizosphere soil has been utilized to detect the soil bacterial community diversity whereas we collected the soil between root zoon and the connection part of the two crops for our sampling method could take into account both root exudates and soil. Second, our work was the first time to reveal the below-ground interactions between legume (peanut) and fiber (cotton) crop species, thus, this interspecific interactions might be different from the previous reports. Therefore, it is expected that the profound role of this intercropping system could be attributed to the changes of the bacterial community composition rather than diversity. To further unravel the role of interspecific interactions, PCoA analysis was carried out and IC, NC-P, and NC-C exerted a distinct difference as compared to MP, MC, SC-P, and SC-C in both 0–20 and 20–40 cm of the top soil at podding stage (Figs. 2e & 3e). Consistent with the data of plant nutrient accumulation (Table 7), these results could be interpreted by the application of root barriers and clearly showed that permeable (100 μm) nylon mesh root barrier performed better than impermeable (solid) root barrier, thus, further confirmed the importance of the below-ground interactions in this intercropping system. The PCoA analysis further indicated that the changes of bacterial community composition at podding stage might play a more vital role in overyielding and enhancing economic returns of this intercropping system. The heatmap based KEGG analysis found that some crucial metabolic pathways such as lipoic acid metabolism, fatty acid biosynthesis, and protein export were significantly enriched in IC, NC-P, and NC-C compared with other treatments (Figs. 2f & 3f). Lipoic acid has been reported to recover metabolic distortions through modulating ion homeostasis such as K^+^ [74] which was consistent with our findings that intercropping significantly accelerated the allocation of K from soil to plants, and then from vegetative tissues to the reproductive organs especially in cotton. Similarly, the facilitation of fatty acid biosynthesis by intercropping would also provide basis for the growth and survival of soil bacteria [75, 76] which in turn promoted the seed cotton yield; however, further investigation are still needed to explore the detailed mechanisms concerning the reduction of peanut pods yield in this intercropping system.

## Conclusions

In summary, the present study leads us to conclude that peanut/cotton intercropping system could induce the seed cotton yield while reduce the peanut pods yield compared with their monocultures. Moreover, the economic returns have been induced under both normal and salinity soil conditions. Using root barrier treatments, we found that the bacterial community composition between monoculture and intercropping were dramatically changed at peanut podding stage and blossom-needling stage. The changes of soil microbial communities could further attribute to the accumulation of soil nutrients such as N, P, and K in both crop species, promotion of the translocation of these nutrients from vegetative tissues to reproductive organs, and finally contribution to the enhanced economic returns. Our findings fill a gap of knowledge on the below-ground interactions in peanut/cotton intercropping system and propose a previously unidentified mechanism combining crop nutrient allocation with soil bacterial community composition. Additionally, this study provides guidance for farmers to select the best cropping system under various soil conditions. Future work could be extended by evaluating the soil bacterial community structures in salinity soil and carrying out multiple year experiments to gain a more general understanding of the microbial-driven below-ground processes in this intercropping system.

## Supplementary Information

**Additional file 1.**

## Data Availability

The datasets generated during and/or analyses during the current study are available in the National Center for Biotechnology Information (NCBI) Sequence Read Archive (SRA) database under Submission ID of SUB10633613 and Accession of PRJNA779843.

## Abbreviations

ANOVA: Analysis of variance
DNA: Deoxyribonucleic acid
IC: Intercropping
K: Potassium
KEGG: Kyoto Encyclopedia of Genes and Genomes
LER: Land equivalent ratio
LERc: Land equivalent ratio of cotton
LERp: Land equivalent ratio of peanut
MC: Monoculture of cotton
Mc: The yields of cotton in monocropping systems
Mp: The yields of peanut in monocropping systems
MP: Monoculture of peanut
N: Nitrogen
NCBI: National Center for Biotechnology Information
NC-C: Permeable root barrier treatments cotton side
NC-P: Permeable root barrier treatments peanut side
SC-C: Impermeable root barrier treatments cotton side
SC-P: Impermeable root barrier treatments peanut side
SRA: Sequence Read Archive
P: Phosphorus
PCR: Polymerase chain reaction
PCoA: The principal coordinates analysis
Yc: The yields of cotton in intercropping systems
Yp: The yields of peanut in intercropping systems
