# Supplementary material for: Peanut and cotton intercropping increases productivity and economic returns through regulating plant nutrient accumulation and soil microbial communities
Source: BMC Plant Biol. 2022 Mar 16;22:121. doi: 10.1186/s12870-022-03506-y (PMC8925217; doi:10.1186/s12870-022-03506-y)
Supplement: Supplementary file 1 — Additional file 1. [file 12870_2022_3506_MOESM1_ESM.docx]

**Table S1** Soil bacterial community diversity parameters of different treatments in various top soils at blossom-needling and podding stages.

| Growth stage/Soil | Treatment | Number of OTUs | Shannon index | Simpson index | ACE | Chao |
| --- | --- | --- | --- | --- | --- | --- |
| Blossom-needling stage/  0-20 cm | MP | 6523.67±436.21 a | 10.49±0.05 a | 0.998136±0.000145 a | 8044.40±634.61 a | 8201.17±656.1 a |
|  | MC | 5532.33±297.49 bc | 10.45±0.05 a | 0.998143±0.000098 a | 6849.17±377.67 bc | 7046.22±348.52 ac |
|  | IC | 6065.33±362.71 ab | 10.42±0.02 a | 0.998041±0.000032 a | 7563.08±471.71 ab | 7697.10±479.81 ab |
|  | NC-P | 5558.00±202.51 bc | 10.31±0.05 a | 0.997899±0.000133 a | 6821.02±290.58 bc | 6941.47±242.47 bc |
|  | NC-C | 5632.33±174.61 bc | 10.51±0.04 a | 0.998213±0.000048 a | 6944.51±188.87 ac | 7175.97±153.77 ac |
|  | SC-P | 5014.67±420.01 c | 10.15±0.38 a | 0.996468±0.002589 a | 6270.85±518.59 c | 6471.39±581.21 c |
|  | SC-C | 5844.33±24.09 abc | 10.52±0.1 a | 0.998182±0.000175 a | 7248.76±35.12 abc | 7428.07±59.9 abc |
| Blossom-needling stage/  20-40 cm | MP | 4514.33±347.63 ab | 10.33±0.02 ab | 0.998125±0.000101 a | 5539.60±474.98 ab | 5703.46±479.15 ab |
|  | MC | 4418.33±221.86 ab | 10.09±0.06 b | 0.997059±0.000156 b | 5418.85±264.83 ab | 5594.07±251.53 ab |
|  | IC | 4814.67±605.8 ab | 10.26±0.05 ab | 0.997843±0.0001 a | 6096.93±890.67 ab | 6234.92±898.5 ab |
|  | NC-P | 4382.00±19.52 ab | 10.24±0.06 ab | 0.997842±0.000137 a | 5433.60±79.25 ab | 5568.65±71.01 ab |
|  | NC-C | 5216.33±36.2 a | 10.45±0.04 a | 0.998019±0.000197 a | 6459.21±88.86 a | 6629.25±84.22 a |
|  | SC-P | 4072.67±161.75 b | 10.22±0.19 b | 0.997823±0.000434 a | 5134.73±221.15 b | 5200.36±269.1 b |
|  | SC-C | 4551.33±277.63 ab | 10.31±0.04 ab | 0.997798±0.000062 a | 5722.50±380.83 ab | 5914.76±349.06 ab |
| Podding stage/  0-20 cm | MP | 4655.67±65.58 cd | 10.25±0.04 ab | 0.9976±0.000153 ab | 5960.51±99.51 cd | 6181.36±119.11 cd |
|  | MC | 4979.67±441.36 cd | 10.27±0.10 ab | 0.997481±0.000123 ab | 6250.13±547.38 cd | 6432.91±598.01 bcd |
|  | IC | 5875.33±158.88 ab | 10.30±0.07 ab | 0.996792±0.000418 b | 7408.60±244.74 ab | 7443.92±246.59 ab |
|  | NC-P | 5398.00±383.12 abc | 10.14±0.069 b | 0.996865±0.000598 b | 6944.07±598.61 ac | 7034.75±552.03 ac |
|  | NC-C | 6122.00±506.97 a | 10.38±0.17 ab | 0.997288±0.000444 ab | 7789.71±617.64 a | 7943.78±576.33 a |
|  | SC-P | 4480.67±158.88 d | 10.18±0.08 ab | 0.997418±0.000458 ab | 5641.40±224.13 d | 5766.95±165.61 d |
|  | SC-C | 5058.33±169.14 bcd | 10.41±0.08 a | 0.997995±0.000142 a | 6385.74±192.56 bcd | 6553.96±191.82 bcd |
| Podding stage/  20-40 cm | MP | 4760.33±631.39 a | 10.32±0.02 ab | 0.997969±0.000139 a | 5929.39±860.45 a | 6085.00±841.38 a |
|  | MC | 4709.00±162.23 a | 10.32±0.17 ab | 0.997886±0.000428 a | 5823.80±194.52 a | 5999.15±154.54 a |
|  | IC | 4588.67±549.72 a | 10.08±0.17 b | 0.997488±0.000264 a | 5847.4±663.98 a | 5990.71±687.99 a |
|  | NC-P | 4875±82.15 a | 10.24±0.13 ab | 0.997789±0.000269 a | 5992.8±121.52 a | 6196.62±171.33 a |
|  | NC-C | 5663±465.06 a | 10.42±0.05 a | 0.997876±0.000017 a | 6982.46±529.86 a | 7235.68±474.3 a |
|  | SC-P | 4270±868.19 a | 10.18±0.08 ab | 0.997805±0.00023 a | 5345.42±1101.15 a | 5521.4±1056.34 a |
|  | SC-C | 4756.33±583.06 a | 10.38±0.11 ab | 0.998123±0.000093 a | 6093.6±918.52 a | 6329.69±840.02 a |

Means denoted by different letters within the same column indicate significant differences according to Tukey’s test (*P* < 0.05); MP: monocropping of peanut; IC: intercropping of peanut and cotton without barriers; NC: intercropping of peanut/cotton with 100 μm nylon mesh barrier, NC-P: peanut strip, NC-C: cotton strip; SC: intercropping of peanut/cotton with solid barrier, SC-P: peanut strip, SC-C: cotton strip.


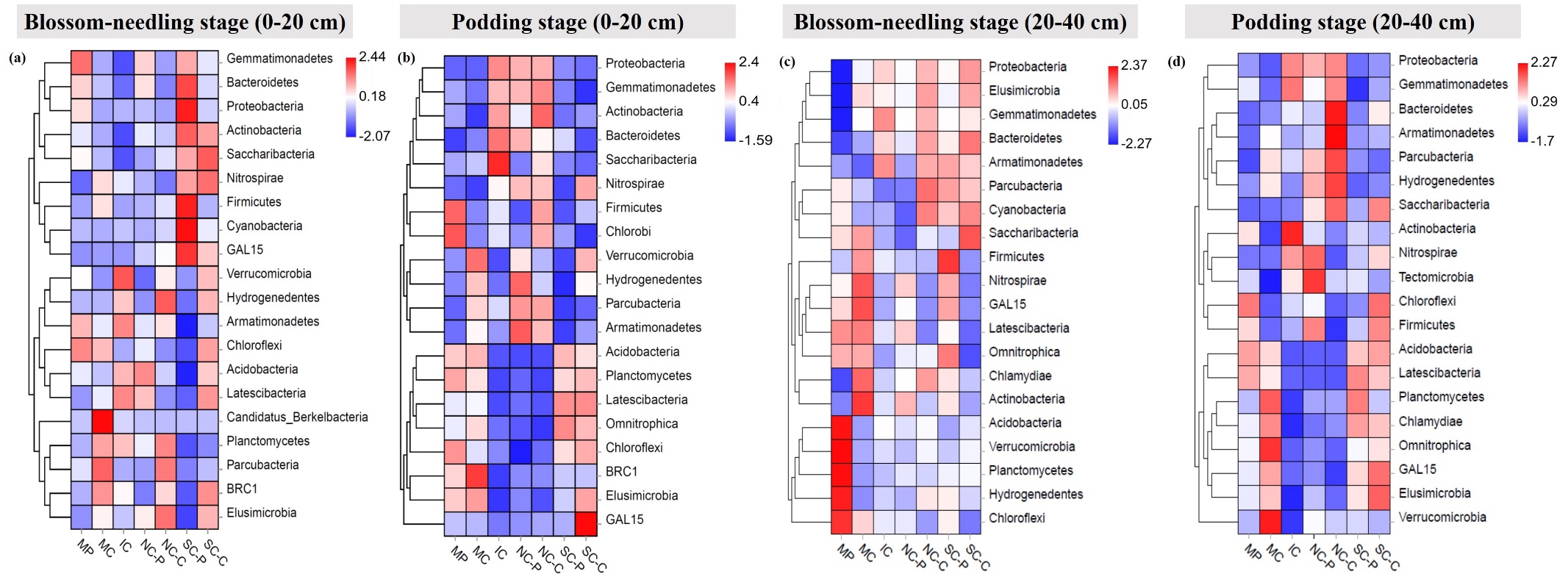


**Fig. S1** The correlation analysis between relative abundance of dominant bacterial genera with different treatments of 0-20 cm (**a**) and 20-40 cm (**c**) at blossom-needling stage, and 0-20 cm (**b**) and 20-40 cm (**d**) at podding stage. The positive and negative correlations are displayed in blue and red color, respectively, while color density represents strength of correlations.
